# Topical Agents for Nonrestorative Management of Dental Erosion: A Narrative Review

**DOI:** 10.3390/healthcare10081413

**Published:** 2022-07-28

**Authors:** Darren Dhananthat Chawhuaveang, Ollie Yiru Yu, Iris Xiaoxue Yin, Walter Yu Hang Lam, Chun Hung Chu

**Affiliations:** Department of Restorative Dental Sciences, Faculty of Dentistry, The University of Hong Kong, Hong Kong SAR, China; dhanant@connect.hku.hk (D.D.C.); irisxyin@hku.hk (I.X.Y.); retlaw@hku.hk (W.Y.H.L.); chchu@hku.hk (C.H.C.)

**Keywords:** tooth erosion, fluorides, anti-erosive agents

## Abstract

A nonrestorative approach to the management of dental erosion is the foremost option: controlling dental erosion. The objectives of this study are to provide an overview and to summarise the effects and properties of topical anti-erosive agents as a nonrestorative treatment of dental erosion. A literature search was conducted on five databases of peer-reviewed literature—Cochrane Library, EMBASE, PubMed, Scopus and Web of Science—to recruit articles published between 1 January 2000 and 31 December 2021. The literature search identified 812 studies; 95 studies were included. Topical anti-erosive agents can be broadly categorised as fluorides, calcium phosphate-based agents, organic compounds and other anti-erosive agents. In the presence of saliva, fluorides promote the formation of fluorapatite on teeth through remineralisation. Calcium phosphate-based agents supply the necessary minerals that are lost due to the acid challenge of erosion. Some organic compounds and other anti-erosive agents prevent or control dental erosion by forming a protective layer on the tooth surface, by modifying salivary pellicle or by inhibiting the proteolytic activity of dentine collagenases. Topical anti-erosive agents are promising in managing dental erosion. However, current evidence shows inconsistent or limited results for supporting the use of these agents in clinical settings.

## 1. Introduction

Dental erosion is the loss of minerals from the dental hard tissue caused by non-bacterial acids [1]. This chemical process often occurs with mechanical wear, such as abrasion and attrition, and is known as erosive tooth wear [1,2]. Erosive tooth wear damages the tooth structure, negatively affecting the aesthetics and the function of the natural dentition [1,2,3]. The loss of tooth structure with opening dentinal tubular often initiates dentine hypersensitivity, which can result in severe, persistent pain and discomfort [2,3]. With the increased consumption of acidic food and beverage, the prevalence of dental erosion has increased significantly over the past few decades [1,4,5]. The estimated global prevalence of dental erosion in primary dentition ranged from 30% to 50% and from 20% to 45% in permanent dentition [6]. Almost all middle-aged populations and more than half of children and adolescents are affected by dental erosion [7]. Therefore, dental erosion has a long-term impact on the oral and general health of the population [2,3].

Restorative treatment for dental erosion can be challenging, invasive and extensive [8,9]. Therefore, it is imperative to identify dental erosion at an early stage, and nonrestorative management aiming for preventive care should be provided [1,10]. Nonrestorative approaches to dental erosion commonly include dietary analysis and counselling, oral health education and topical use of anti-erosive agents [5,11,12]. Dietary analysis and counselling are important for preventing excessive consumption of acidic beverages and foodstuffs that potentially damage dentition [1,10]. Oral health education is essential to promoting the protective factors that effectively prevent dental erosion [10,13]. Among the various nonrestorative approaches, topical anti-erosive agents are commonly used to manage dental erosion [11,12].

Although nonrestorative approaches should be prioritised in managing dental erosion, anti-erosive agents for preventing and managing dental erosion have never been elucidated in the literature. Therefore, this narrative review aims to provide an overview and to summarise the effects and properties of topical anti-erosive agents as a nonrestorative treatment of dental erosion.

## 2. Anti-Erosive Agents in the Literature

**Figure 1:**
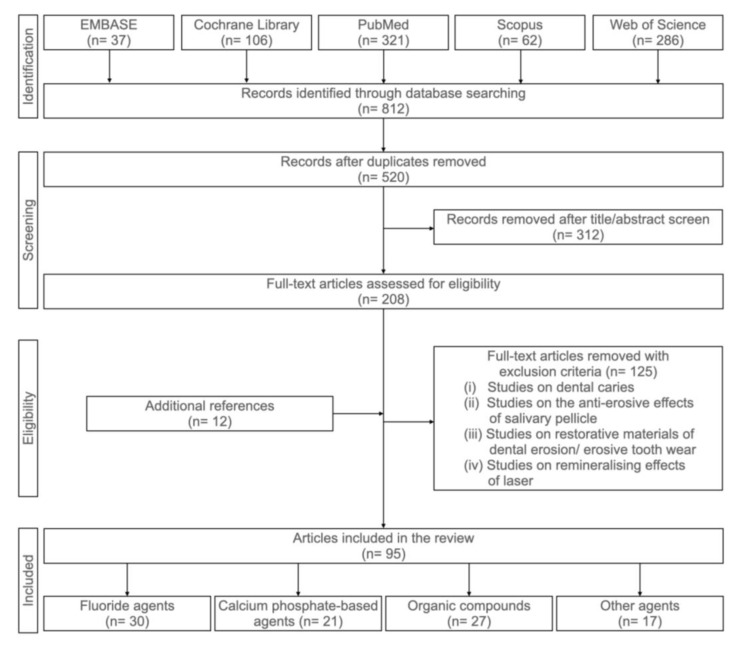
Flowchart summarising the reference-selection process.

**Figure 2:**
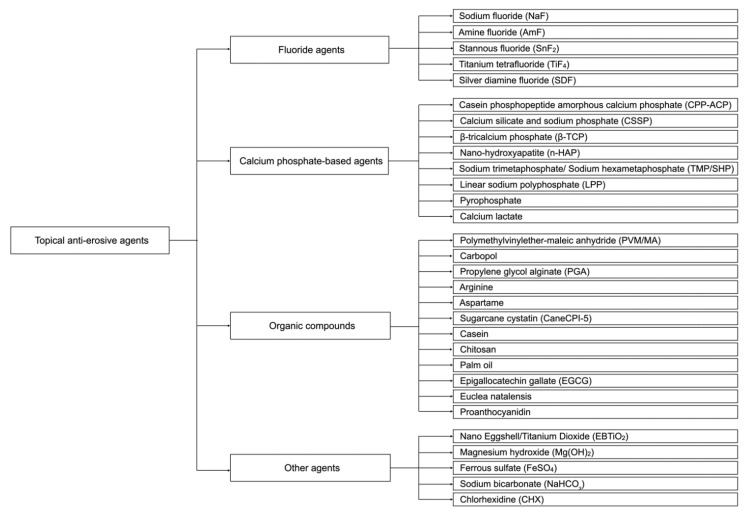
Topical anti-erosive agents.

The literature search of this narrative review was conducted on five databases: Cochrane Library, EMBASE, PubMed, Scopus and Web of Science, to recruit articles published between 1 January 2000 and 31 December 2021. The keywords used in the search were (“Dental erosion” OR “Tooth erosion” OR “Enamel erosion” OR “Dentine erosion” OR “Dentin erosion” OR “Eroded” OR “Erosive lesion” OR “Erosive demineralisation*” OR “Erosive tooth wear”) AND (“Prevention” OR “Protection” OR “Protective effect*” OR “Anti-erosion” OR “Anti-erosive”). Figure 1 shows a flowchart of our systemic search of the literature. The inclusion criteria were (i) studies that assessed the anti-erosive effects of topical agents and (ii) studies published between 1 January 2010 and 30 September 2021. The exclusion criteria were (i) studies on dental caries, (ii) studies on the anti-erosive effects of salivary pellicle, (iii) studies on the restorative management of dental erosion/erosive tooth wear and (iv) studies on the remineralising effects of carbon dioxide laser. Topical anti-erosive agents can be broadly categorised as topical fluorides, calcium phosphate-based agents, organic compounds and other anti-erosive agents (Figure 2).

### 2.1. Fluoride Agents

Fluoride agents are the most common topical anti-erosive agents [5] and include sodium fluoride (NaF), amine fluoride (AmF), stannous fluoride (SnF_2_), titanium tetrafluoride (TiF_4_) and silver diamine fluoride (SDF). They are divided into two groups: conventional monovalent fluoride, such as NaF and AmF [5], and polyvalent fluoride with metal cations, such as TiF_4_, SnF_2_ and SDF [5,11,14]. Topical fluoride facilitates the formation of a protective layer on the tooth surface and enhances the acid resistance of dental hard tissue [14,15]. Some fluoride agents can modify salivary pellicle and enhance the acid resistance of salivary pellicle [16,17]. Fluoride can also inhibit the activation of matrix metalloproteinases (MMPs) in the dentine matrix [14,15].

#### 2.1.1. Sodium Fluoride (NaF)

NaF is a conventional monovalent fluoride [5]. There are plenty of commercially available NaF products [18,19,20,21]. Over the counter, NaF toothpaste contains up to 1450 ppm fluoride [5,20]. NaF toothpaste with 5000 ppmF is also available as a prescription [5,22]. In addition, 5% NaF varnish containing 22,600 ppmF is a commonly used professional fluoride agent [5,20]. NaF application leads to a calcium fluoride-like layer (CaF_2_) forming on the tooth surface [5,22]. This layer is a protective barrier that blocks acid diffusion through the tooth’s surface [23]. CaF acts as a reservoir of calcium and fluoride ions to enhance the remineralisation process of the hard tissue [5,22]. Sodium ions may interact with the salivary proteins in the acquired salivary pellicle [12,24]. It improves the re-adsorption of proteins on the eroded tooth surface and may enhance the erosive resistance of salivary pellicle [24]. Moreover, NaF is an MMP inhibitor that inhibits MMP activation [25] and maintains the demineralised organic matrix (DOM), which acts as a barrier to acid diffusion on the dentine surface [25,26].

NaF reduced surface loss, mineral loss and microhardness change in enamel specimens after erosive challenges [20,27,28]. However, some studies reported that the anti-erosive effects of NaF are limited [5,12,23,29,30].

#### 2.1.2. Amine Fluoride Containing Agents (AmF)

Products containing AmF are available in different forms such as toothpaste, mouthrinse and solution [31,32,33,34,35]. In collaboration with the University of Zurich, a company (GABA, Schweiz, Colgate-Palmolive, Therwil, Switzerland) developed and patented AmF with NaF and SnCl_2_ [12,36]. Using organic molecules as carriers, they demonstrated that AmF significantly reduced the solubility of the enamel and thereby increased the resistance of enamel against acid attack [12,36]. AmF provide CaF_2_ precipitates as a protective layer on the tooth surface [12,37]. Moreover, AmF have a pronounced affinity regarding enamel by raising the quantity of fluoride in the saliva [37].

AmF combined with SnCl_2_ or SnF_2_ reduced human enamel or dentine loss after erosive challenges [12,38]. AmF alone decreased calcium ion release in human dentine specimens in acid challenges [39], but the protective effect was limited [34,35,40].

#### 2.1.3. Stannous Fluoride Containing Agents (SnF_2_)

Anti-erosive products using SnF_2_ or SnCl_2_ with fluoride as active ingredients are available on the market [41,42,43]. The stannous ions worked synergistically with fluoride and enhanced the anti-erosive effects of fluoride [15,44,45]. Stannous ions in these products have a higher affinity for mineral contents than organic contents [15]. These stannous ions interact with hydroxyapatite and fluoride and form a layer of Sn_2_OHPO_4_, Sn_3_F_3_PO_4_, Ca (SnF_3_)_2_ and CaF_2_ on the tooth surface [36,46,47,48,49]. This layer is more stable and acid-resistant than the layer formed after NaF application alone [15,36,46]. Moreover, stannous ions interact with salivary proteins in acquired salivary pellicle [16,24] and enhance the acid resistance of salivary pellicle [16,17]. Stannous ions can also inhibit the activation of MMPs in the dentine matrix and prevent the organic matrix of the demineralised dentine surface from degrading [50].

SnF_2_ reduced surface loss, decreased calcium ions release and decreased the microhardness change in enamel specimens after erosive challenge [24,33,51,52]. Nevertheless, the protective layer of SnF_2_ is not stable at neutral or alkaline conditions [15]. It should be noted that SnF_2_/SnCl_2_ may cause staining on the dental hard tissue with prolonged use [32].

#### 2.1.4. Titanium Tetrafluoride (TiF_4_)

No commercially available product of TiF_4_ is currently available. TiF_4_ reacts with the hydroxyapatite on the tooth surface and forms a glaze-like layer of titanium dioxide (TiO_2_) and hydrated titanium phosphate (TiPO_4_) [50,53,54]. The TiO_2_ and TiPO_4_ layer coats the tooth surface and acts as a protective layer against erosive challenges [53,54]. Furthermore, this layer promotes fluoride uptake to the tooth surface [50,53] and increases the CaF_2_ deposits on the tooth surface [55]. The interaction of TiF_4_ and fluorapatite was found in the subsurface area in the demineralised enamel [54,56]. This layer is more acid-resistant than the CaF_2_ layer formed after NaF application [53].

TiF_4_ reduced human dentine loss, decreased microhardness change in human enamel specimens and occluded human dentinal tubule in erosive attacks [50,57,58,59]. However, the extreme acidity of the TiF_4_ solution may weaken its anti-erosive effect [60,61]. The interaction with saliva also reduces the protective effect of TiF_4_, which is unavoidable in the oral cavity [54,62]. Another concern is that TiF_4_ can cause reversible staining on the tooth surface [50,63,64].

#### 2.1.5. Silver Diamine Fluoride (SDF)

SDF is an alkaline agent (pH ~10) [65]; 38% SDF is a solution that contains a high concentration of fluoride and silver ions [65,66]. Several SDF products are available on the market [66,67]. SDF application facilitates the deposition of silver compounds on the tooth surface and may act as a protective layer against dental erosion [16,67]. SDF reduces demineralisation and promotes remineralisation on the tooth surface by promoting the deposition of CaF_2_ and the formation of fluorapatite crystals on the tooth surface [16,65,68]. SDF can also inhibit MMPs [69] and may decrease the erosive demineralisation of dentine [69].

SDF has been widely investigated in anti-caries activity but the evidence of SDF on managing dental erosion is insufficient [70]. SDF decreased bovine enamel and dentine loss after erosive challenges [16]. A main disadvantage of SDF is the permanent black staining on the tooth surface [66,69,70].

### 2.2. Calcium Phosphate-Based Agents

Calcium phosphate-based agents supply the necessary minerals that are lost due to the acid challenge of erosion [71]. Calcium phosphate-based agents include casein phosphopeptide amorphous calcium phosphate (CPP-ACP), calcium silicate sodium phosphate (CSSP), β-tricalcium phosphate (β-TCP), nano-hydroxyapatite (n-HAP), sodium trimetaphosphate/sodium hexametaphosphate (TMP/SHP), linear sodium polyphosphate (LPP), pyrophosphate and calcium lactate.

#### 2.2.1. Casein Phosphopeptide Amorphous Calcium Phosphate (CPP-ACP)

CPP-ACP is used in oral health products such as varnish, cream, chewing gum and acidic drinks for the management of dental caries [11]. The addition of fluoride to CPP-ACP results in the formation of casein phosphopeptide amorphous calcium fluoride phosphate (CPP-ACFP). CPP-ACP comprises a high amount of calcium and phosphate ions [36,49]. These ions form a calcium hydrogen phosphate precipitation layer [49], which acts as a physical barrier between acids and tooth surfaces [72,73,74]. Notably, the precipitation layer showed non-homogeneous distribution and may be easily detached by acids [73,75]. Calcium and phosphate ions are maintained around the tooth surface even under acidic conditions and reduce demineralisation process [11,36,49]. It also promotes remineralisation by supplying the mineral ions on the eroded surface [11,36]. In addition, CPP-ACP can modify the acquired salivary pellicle by increasing the electron-dense layer of the pellicle [73,76].

In situ studies revealed that CPP-ACP reduced the surface loss, microhardness change and calcium ions release in human enamel specimens [73,77,78,79]. Similarly, in situ studies of CPP-ACFP reported that CPP-ACFP reduced bovine enamel loss [80] and decreased microhardness change in human enamel after erosive cycles [81]. The effectiveness of CPP-ACP depends on the concentration, the vehicles and time of application [73,74,75]. The optimal time of application of CPP-ACP is contradictory in the literature [73,82]. A previous study showed that CPP-ACP had a limited working time [73].

#### 2.2.2. Calcium Silicate and Sodium Phosphate (CSSP)

CSSP has been used as an additive in oral hygiene products for many years [83]. CSSP provides calcium and phosphate ions and raises the concentration of the mineral ions surrounding the tooth surface to saturated levels [36,83]. Thus, it helps reduce the demineralisation on the tooth surface [32,36] and promotes the remineralisation process of erosive dental hard tissue [32,36,84]. The CSSP also forms a protective layer by depositing calcium silicate particles on the tooth surface [32,83].

No study has investigated the effect of CSSP alone. In situ studies of CSSP reported that CSSP reduced bovine enamel loss [83], decreased the human enamel hardness change [85] and decreased dentine permeability after erosive challenges when combined with fluoride [86]. However, the protective effect of CSSP has a relatively short effective time [32]. The protective layer is unstable and can be easily removed by a strong acid [84]. In addition, CSSP may induce demineralisation on the tooth surface by itself due to the high acidity of the agent [32].

#### 2.2.3. β-Tricalcium Phosphate (β-TCP)

β-TCP is a bioactive agent mainly comprising calcium and phosphate [71]. β-TCP provides calcium and phosphate ions to the tooth surface [71]. The calcium and phosphate ions maintain saturation levels around the tooth surface and induce mineral deposition on the tooth surface [71,87]. Moreover, β-TCP provides nucleation of the remineralisation process [71,87]. This nucleation facilitates the remineralisation of the eroded surface [71].

No study has investigated the anti-erosive effect of β-TCP alone. Previous in situ studies of β-TCP revealed that β-TCP reduced calcium and phosphate ion release, surface loss and hardness change in human enamel specimens when used with fluoride [71,87,88,89].

#### 2.2.4. Nano-Hydroxyapatite (n-HAP)

n-HAP or nano-sized zinc-carbonate-hydroxyapatite is a synthetic hydroxyapatite, of which its size is approximately 20–100 nm [90,91,92]. The common concentration of n-HAP ranges between 1 and 10% [90]. n-HAP chemically binds to the natural apatite on the tooth structure and forms a crystalised apatite layer [90,93]. In addition, n-HAP can release calcium ions to maintain the mineral ions at a high level around the tooth surface and oral environment, which decrease demineralisation and increase remineralisation [90,94]. n-HAP can also be a template for crystal growth and remineralisation [93,95].

One in situ study reported that n-HAP decreased the human enamel hardness change after erosive challenge [90]. However, it was also reported that n-HAP had limited preventive effects on dental erosion [90].

#### 2.2.5. Sodium Trimetaphosphate/Sodium Hexametaphosphate (TMP/SHP)

TMP and SHP are inorganic polyphosphate compounds [29,96]. TMP is usually used as an additive in fluoride varnish, toothpaste and mouthrinse, whereas SHP is mainly added to fluoride toothpaste [14,29,49]. TMP can provide an acid-resistant layer by adsorbing onto the hydroxyapatite structure of the tooth surface and the collagen of the dentine surface [29,97,98,99]. The phosphate structures in the protective layer can incorporate the CaF_2_ layer due to fluoride application [98]. SHP has a similar action to TMP on the tooth surface [14]. Additionally, SHP may infiltrate into the demineralised organic contents at the dentine surface and facilitates the formation of a scaffold for remineralisation [14]. SHP also enhances the level of acid-resistant salivary proteins in the acquired salivary pellicle [49,97].

No study has investigated the anti-erosion effect of TMP or SHP alone. In situ studies demonstrated that TMP or SHP reduced surface loss and decreased surface microhardness change in bovine enamel when combined with fluoride [100,101]. A low concentration of TMP/SHP worked synergistically with fluoride [99], while a high concentration of TMP/SHP may reduce the anti-erosive effect of fluoride [100].

#### 2.2.6. Linear Sodium Polyphosphate (LPP)

LPP is a long-chain polyphosphate agent commonly used as the additive of non-alcoholic drinks [102,103]. LPP has phosphate groups that can bind with positively charged particles on the tooth structure [102,103] and forms an acid-resistant layer [103,104]. The LPP also works synergistically with fluoride and stannous ions in controlling dental erosion [102,103].

LPP decreased bovine enamel loss in in situ erosive challenges when combined with fluoride [104]. Likewise, in vitro studies demonstrated that LPP decreased surface loss and hydroxyapatite dissolution in bovine enamel and dentine when combined with SnCl_2_ and fluoride [102,103]. LPP is more effective on enamel than on dentine because dentine has fewer binding sites for LPP [102]. However, some studies showed that LPP might compete with the anti-erosive protein in the salivary pellicle [104] or fluoride [105] for the binding site on the tooth surface and decrease their protective effect against dental erosion [104,105].

#### 2.2.7. Pyrophosphate

Pyrophosphate or phytate is an organic polyphosphate, which is mainly found in cereals and seeds [106]. It is a cyclic structure with six phosphate groups without direct phosphate–phosphate bonds [105,106]. The pyrophosphate rapidly adsorbs and multi-point binds with hydroxyapatite of the tooth surface [105]. Moreover, it inhibits the diffusion of the ions between acids and the tooth surface [105,106]. Thus, pyrophosphate reduces erosive demineralisation [105,106].

One in situ study reported that pyrophosphate completely inhibited the remineralisation property of fluoride [105]. It may also compete with other mineral ions and inhibit them from binding to the tooth surface [105].

#### 2.2.8. Calcium Lactate

Calcium lactate or calcium effervescent tablets have been used to prevent the softening of dental hard tissue caused by erosive drinks [28,48]. Calcium lactate mouthrinses or solutions provide extra calcium ions in the saliva [28,48] and reduce the demineralisation of the tooth during acid attacks [48]. Moreover, calcium ions may react with the fluoride in the oral cavity and increase CaF deposition on the tooth surface [28,107]. The CaF acts as a fluoride and calcium ion reservoir and promotes remineralisation [93,95].

An in vitro study reported that calcium lactate with fluoride decreased bovine enamel loss after erosive challenges [107]. However, an in situ study reported that a calcium lactate-containing solution could not prevent enamel erosion [48].

### 2.3. Organic Compounds

Anti-erosive organic compounds include polymer agents, such as polymethylvinylether-maleic anhydride (PVM/MA), carbopol and propylene glycol alginate (PGA), and these agents are derived from animals or plants’ carbohydrates; lipids; or proteins, such as arginine, aspartame, sugarcane cystatin (CaneCPI-5), casein, chitosan, palm oil, epigallocatechin gallate (EGCG), Euclea natalensis plants and proanthocyanidin.

#### 2.3.1. Polymethylvinylether-Maleic Anhydride (PVM/MA)

PVM/MA is a film-forming polymer, which is commonly used as an additive in beverages [108]. It is added to oral health products to control dental erosion [108]. PVM/MA binds to the mineral ions in the enamel and forms a protective layer [108,109]. It also binds to mineral ions and type I collagen in the dentine [110]. However, the protective layer formed by PVM/MA could easily be removed by acids because of the weak bond of PVM/MA with the tooth [108]. PVM/MA also helps with fluoride retention on the tooth surface and sustains fluoride release for a longer period [109,110,111].

An in situ study reported that PVM/MA enhanced surface hardness on bovine enamel after erosion compared with the control group [111]. Previous in vitro studies showed that PVM/MA positively interacted with fluoride and reduced surface loss in human and bovine dentine specimens [108,110,111].

#### 2.3.2. Carbopol

Carbopol is a high molecular weight polymer with a negatively charged centre, which allows it to chelate with calcium ions [40,112]. Carbopol strongly binds with calcium ions [17,40] and forms a protective layer that covers the tooth surface [40,112]. Some studies have demonstrated that carbopol enhanced fluoride adsorption and promoted fluoride retention in the oral cavity [40,112].

Studies reported that carbopol with fluoride reduced enamel loss, increased enamel hardness and decreased the hydroxyapatite dissolution in erosive challenges [40,112]. However, the acidity (pH = 2.7–3.3) of carbopol may intensify the dissolution of dental hard tissue [40]. Carbopol also competes with salivary proteins for the binding sites on the tooth surface and may interfere with the anti-erosive potential of the acquired salivary pellicle [17].

#### 2.3.3. Propylene Glycol Alginate (PGA)

PGA is a natural polymer derived from brown seaweeds that is commonly used in the food and biomedical industry, such as emulsifiers, stabilisers and thickening agents [108,113]. It has low toxicity, low cost, and high viscosity and biocompatibility [108,113]. The carboxylic groups of the PGA can bind to the calcium ions on the tooth surface and provides a protective layer [108,113]. The anti-erosive effect of the PGA is affected by the hydrogen ion level in acidic solutions because the hydrogen ions bind with the carboxylic groups of PGA and change the number of available carboxylic groups in PGA that can bind to calcium ions on the tooth surface [113]. PGA also competes with salivary proteins in salivary pellicle for binding sites on the tooth surface [113]. PGA with fluoride reduced bovine enamel and dentine loss after erosive challenge [108,113]. Notably, PGA alone cannot protect the tooth surface from erosion [108,113].

#### 2.3.4. Arginine

Arginine is a positively charged amino acid that has a strong affinity to the dentine surface [56,114]. It improves the attachment of calcium to the tooth surface and promotes calcium carbonate precipitation [49,115,116]. This precipitation layer enhances the resistance of dental hard tissue to acids [115,116]. The precipitation layer helps to occlude dentinal tubules and alleviates dentine hypersensitivity [114,115].

The combined application of arginine with fluoride or calcium carbonate showed a positive result in controlling erosion [114,116,117]. The previous in situ studies demonstrated that arginine with fluoride decreased dentine permeability to acids by occluding human dentinal tubule [114,118]. In vitro studies reported that arginine reduced the hardness change and surface loss on bovine enamel [56,116].

#### 2.3.5. Aspartame

Aspartame is a synthetic dipeptide and an artificial non-saccharide sweetener [119]. It is widely used as a sugar substitute in food and drink products [119]. Aspartame can be degraded to phenylalanine, aspartic acid and methanol [119]. Phenylalanine contains carboxylic and amino groups that can capture hydrogen ions in the erosive acids and can reduce the acidity of the acids [119]. Hence, it can reduce the demineralisation of the enamel surface [119,120].

A previous study showed that the anti-erosive effect of aspartame is limited [120]. All in situ and in vitro studies that investigated the anti-erosive property of aspartame revealed aspartame had no significant protective effect on the bovine enamel compared to no treatment [119,120].

#### 2.3.6. Sugarcane Cystatin (CaneCPI-5)

CaneCPI-5 is a novel synthesised sugarcane cystatin [121]. It strongly binds to hydroxyapatite of the enamel surface and forms a protective layer [121,122]. The CaneCPI-5 can also improve the protective effect of the salivary pellicle by increasing the number of acid-resistant proteins such as cystatin B [121,123,124]. In addition, CaneCPI-5 inhibits MMPs and reduces the severity of dentine erosion [122,123].

One in situ study revealed that CaneCPI-5 reduced bovine enamel loss in erosive challenge [121]. In vitro studies reported that CaneCPI-5 reduced surface loss in bovine enamel and dentine and hardness change in bovine dentine [122,123].

#### 2.3.7. Casein

Casein is a protein that is commonly found in colostrum or milk products [125,126]. Casein comprises three subfractions: α-, β- and κ-casein [125]. The casein can adsorb onto the hydroxyapatite and forms a protective layer of milk protein on the tooth surface [125,126]. Casein has many amino acid sequences such as phosphoserine, histidine, glutamate and aspartate [126]. These amino acids could act as a buffer to increase pH and decrease demineralisation during acidic conditions [126]. Furthermore, casein can modify the pellicle’s compositions and enhances the protective effects of salivary pellicle [125,126].

Casein reduced calcium and phosphate ion release in human enamel specimens after in situ erosive challenges [125]. Casein reduced surface loss and reduced microhardness change on bovine enamel in in vitro erosive challenges [126].

#### 2.3.8. Chitosan

Chitosan has been used as an additive in some fluoridated oral health products [47]. It increases the viscosity and stability of fluoride-containing agents [55,104] and enhances the anti-erosive capacity of fluoride agents [49,104]. Chitosan alone provides a protective layer on the tooth surface by binding with mineral ions on the tooth surface [14,55]. It can be modified by phosphorus ions and forms phosphorylated chitosan, which promotes the remineralisation process by chelating with calcium and phosphate ions on the tooth surface [127]. The alkaline phosphorylated chitosan inhibits MMP degradation and preserves the integrity of collagen fibrils [127]. Chitosan also modifies the acquired salivary pellicle by increasing the attachment of acid-resistant salivary proteins [47,123,128].

Previous in situ studies supported that chitosan with fluoride could reduce human enamel surface loss in erosive challenges [128,129]. In vitro studies reported that chitosan with fluoride decreased bovine dentine loss [130,131]. Chitosan alone had a weak anti-erosive effect [55,127,131]. It should be noted that chitosan with different pH levels has different anti-erosion effects [127]. Alkaline phosphorylated chitosan is more acid-resistant than neutral or acidic ones [127].

#### 2.3.9. Palm Oil

Palm oil is an edible, safe and low-cost natural product [132,133]. Palm oil has been widely used in foods, cosmetics and medical products [133]. It can modify the composition and ultrastructure of the outer layer of the salivary pellicle and form an acid-resistant hydrophobic lipid-enriched pellicle [132].

Palm oil reduced bovine enamel loss in erosive challenges in an in situ study [132]. An in vitro study reported palm oil decreased bovine enamel hardness change [133].

#### 2.3.10. Epigallocatechin Gallate (EGCG)

EGCG is a type of polyphenol that is found in green tea [134,135]. EGCG forms an acid-resistant precipitation layer by binding with calcium ions on the tooth surface [134]. In addition, EGCG inhibits MMP activity in the dentine matrix and decreases erosive demineralisation in the dentine [134,135,136]. Moreover, EGCG modifies acquired salivary pellicle’s compositions by increasing the number of acid-resistant proteins [137].

The in situ studies revealed that EGCG reduced bovine dentine loss in erosive challenges [26,138]. An in vitro study reported that EGCG decreased human enamel and dentine loss after acid challenges [134]. However, the effectiveness of EGCG gradually reduced after interacting with acids and could not protect the tooth from prolonged erosive attacks [134,135]. One in vitro study also stated that EGCG might have an antagonistic effect with fluoride [134].

#### 2.3.11. Euclea Natalensis Plant Extracts

Euclea natalensis is a plant from Africa [139,140]. The active ingredients of Euclea natalensis extracts are taninus; naphthoquinone; flavonoids; and polyphenolic compounds that demonstrated fungicidal, antibacterial, insecticidal, phytotoxic, cytotoxic and anti-carcinogenic properties [139,140]. Taninus interacts with salivary proteins in the salivary pellicle and forms protein–taninus complexes [140]. Therefore, Euclea natalensis compounds can modify salivary pellicle and form a protective layer on the tooth surface [139,140]. Naphthoquinone is an MMP inhibitor that preserves the organic matrix of the dentine surface in an acid attack [139].

An in situ study reported that Euclea natalensis plant extracts decreased human dentine loss in a 5-day erosive challenge [139]. Euclea natalensis plant extracts also reduced human dentine permeability in an in vitro study [140]. However, Euclea natalensis plant extracts cause temporary light yellow staining on the tooth surface and oral tissues [140]. It should be noted that the effects of the Euclea natalensis plant against erosive demineralisation are scarce in the literature, with only one in situ study and one in vitro study available [139,140].

#### 2.3.12. Proanthocyanidin

Proanthocyanidin is a polyphenol that is commonly found in natural fruits and nuts [141,142]. It has been used in adhesive products in restorative dentistry [139,141]. Proanthocyanidin is effective at inhibiting MMPs 1, 2, 8 and 9 [141,142]. It reduces dentine loss by preserving collagen fibrils and delays acid diffusion [143]. In addition, proanthocyanidin enhances collagen strength by increasing collagen cross-links [144] and forms an insoluble complex that promotes precipitation of calcium ions from saliva [141].

Proanthocyanidin reduced bovine dentine loss in erosive challenges [141,142]. However, the by-products of proanthocyanidin are acidic and decrease the pH of the surrounding tissue [143]. Moreover, proanthocyanidin causes brownish staining in the dentine surface [143].

### 2.4. Other Anti-Erosive Agents

Other anti-erosive agents are agents that cannot be classified into the groups mentioned above, including nano eggshell/titanium dioxide (EBTiO_2_), magnesium hydroxide (Mg(OH)_2_), ferrous sulfate (FeSO_4_) and sodium bicarbonate (NaHCO_3_) and chlorhexidine.

#### 2.4.1. Nano Eggshell/Titanium Dioxide (EBTiO_2_)

EBTiO_2_ is an eggshell-modified agent containing calcium, phosphorus, strontium, zinc, fluoride, copper and titanium ions [95,145]. EBTiO_2_ facilitates the formation of a protective layer of calcium carbonate and TiO_2_ on the tooth surface [145]. The calcium carbonate can also occlude the dentinal tubule and protects dentine from demineralisation [146]. EBTiO_2_ also provides mineral ions on the tooth surface to decrease demineralisation and to promote remineralisation [146].

EBTiO_2_ reduced bovine enamel loss in an in situ erosive challenge [145] and occluded bovine dentinal tubule in an in vitro setting [146]. It should be noted that the calcium carbonate in EBTiO_2_ is unstable in the acidic environment, which limits its use in highly acidic conditions [147]. There are also concerns about the safety and biocompatibility of EBTiO_2_ [146].

#### 2.4.2. Magnesium Hydroxide (Mg(OH)_2_)

Mg(OH)_2_ is an inorganic compound with low solubility and cytotoxicity [148,149]. It is an active component of antacid products for neutralising acidity from gastroesophageal reflux disease [148,149]. Mg(OH)_2_ reacts with hydrogen ions and produces magnesium chloride (MgCl_2_), insoluble magnesium salt and water [148,150]. Mg(OH)_2_ also reacts with calcium ions or calcium carbonate in oral fluids and form a protective layer composed of magnesium carbonate (MgCO_3_) and calcium hydroxide (Ca(OH)_2_) on the tooth surface [148]. Magnesium ions in Mg(OH)_2_ can incorporate into the surface layer of the enamel and modify the enamel crystallographic properties [148,151], which makes the enamel surface stronger [151].

Previous in situ studies reported that Mg(OH)_2_ reduced human enamel loss [150] and human enamel microhardness change after erosive cycles [148,150]. However, Mg(OH)_2_ could not prevent erosion in acidic solutions with a pH lower than 2 [148,150] because Mg(OH)_2_ can easily dissolve in highly acidic conditions [149].

#### 2.4.3. Ferrous Sulfate (FeSO_4_)

FeSO_4_ or iron ion is an inhibitor of MMP-2 and 9 [152,153], which inhibits the hydrolysing of the collagen fibrils in the dentine surface [152,154]. The application of FeSO_4_ on the tooth surface facilitates the formation and precipitation of a protective layer composed of ferric phosphate (FePO_4_) and ferric salts on the tooth surface [154,155].

FeSO_4_ reduced both human and bovine dentine surface loss [152,154] and reduced the microhardness change in the human enamel and dentine after erosive challenges in in situ settings [154]. Nevertheless, FeSO_4_ may cause stains on the tooth surface, particularly when it is used in high concentrations or prolonged periods [152,155].

#### 2.4.4. Sodium Bicarbonate (NaHCO_3_)

NaHCO_3_ has high solubility and can be rapidly dissolved in oral fluid [156,157]. It acts as an acid neutraliser due to its alkalinity [156]. The bicarbonate ions from NaHCO_3_ work synergistically with salivary bicarbonate and increase the buffering capacity of the oral fluid [156,158].

In situ studies demonstrated that NaHCO_3_ reduced the surface loss of bovine enamel [156], but it had no effects on the microhardness change in bovine enamel after erosive challenges [156,157,158]. Hence, the anti-erosive of NaHCO_3_ is limited [157,158].

#### 2.4.5. Chlorhexidine (CHX)

CHX is used in controlling dental erosion because it is an inhibitor of MMPs [141,159,160]. It inhibits collagen degradation by inhibiting MMPs 2, 8 and 9 in acidic attacks [141,159]. In addition, CHX binds with calcium ions and forms a protective precipitation layer on the tooth surface [26].

CHX reduced the surface loss and hardness change in dentine specimens after erosive challenges in in situ settings [159,160,161]. CHX should not be used with fluoride in controlling dental erosion because it has an antagonistic effect with fluoride [161]. It should be noted that prolonged use of CHX may cause tooth discolouration, loss of taste and mucosal irritation [141].

## 3. Discussion

The included studies were conducted with various study protocols and different assessments such as surface loss, microhardness change or surface morphology of the specimens. Due to the heterogeneous research methodology and outcome measures, it was not possible to provide a quantitative assessment of the anti-erosive effects of the included agents in the current study. Therefore, the effects of the included topical anti-erosive agents were summarised without quantitative analysis.

This review introduced four categories of anti-erosive agents for the nonrestorative management of dental erosion, but not all of them are available on the market. The commercially available agents for anti-erosive are shown in Table 1. Fluoride agents revealed the most promising properties in the included studies, especially the combination agents between conventional monovalent and polyvalent fluoride. Unfortunately, more than 2/3 of the included agents are under development. Approximately half of the included studies investigated the remineralising effect of the anti-erosive agents. These studies used erosive challenge to simulate the clinical situation in patients who had been affected by dental erosion. The topical anti-erosive agents were applied on the teeth after erosive challenges. The other half of the studies investigated the preventive effects of the anti-erosive agents, where the anti-erosive agents were applied before the erosive challenge on the teeth. Therefore, the options of topical anti-erosive agents for clinical use are currently limited and the application time of topical anti-erosive is inconclusive.

**Figure 3:**
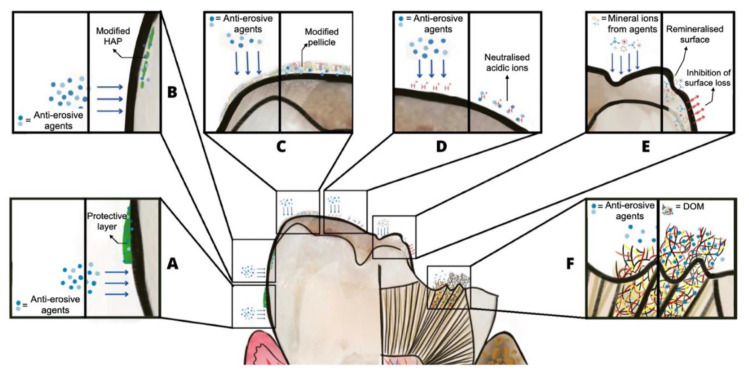
Anti-erosive properties of the topical anti-erosive agents: (**A**) form a protective layer on the tooth surface, (**B**) modify hydroxyapatite, (**C**) modify salivary pellicle, (**D**) neutralise acidic ions, (**E**) supply mineral ions and (**F**) inhibit MMP activation.

Based on previous studies, we have summarised the anti-erosive properties of the topical anti-erosive agents (shown in Figure 3). Most anti-erosive agents can precipitate on the tooth surface and form a protective layer [21,47]. This protective layer reduces the mineral ion exchange between acids and the tooth [103,116]. Some agents such as fluoride and magnesium hydroxide can modify the hydroxyapatite crystal in the tooth and make it more acid-resistant [148,151]. Fluoride agents and most organic compounds can modify the salivary pellicle’s thickness, compositions or ultrastructure and can enhance the anti-erosive capacity of salivary pellicle [16,17,33,76,124]. Some agents are alkaline and can neutralise the acidity of the erosive solutions on the tooth surface [65,149,156]. Calcium phosphate-based agents can supply calcium or phosphate ions that are lost from the tooth surface in acidic conditions [36,71,109,110,111]. Some agents can inhibit MMPs, preserve the demineralised organic matrix of the dentine structure and hamper acid diffusion [83,141,152,154,159,160]. The properties of each anti-erosive agent are shown in Table 2.

Future research should focus on enhancing the anti-erosive effects of topical anti-erosive agents. Most topical anti-erosive agents are used to prevent dental erosion or to control dental erosion in the early stages. The effectiveness of these agents on the management of advanced dental erosion or erosive tooth wear is not satisfactory. The effects of the topical anti-erosive agents may be increased by adding one or more anti-erosive properties. Favourable properties of anti-erosive agents includes forming a protective layer on the tooth surface, modifying hydroxyapatite crystal, modifying salivary pellicle, supplying calcium/phosphate ions, neutralising the acidity and inhibiting MMP activation (shown in Figure 3). No topical anti-erosive agents have all of these favourable properties. Therefore, developing novel agents with most or all the properties and enhanced anti-erosive effects are essential for the nonrestorative management of dental erosion. 

Clinical studies with higher evidence levels are needed to prove the effectiveness of these topical anti-erosive agents. Although several of the anti-erosive agents provided promising results in protecting the tooth from dental erosion, it should be noted that most studies of anti-erosive agents were in vitro studies or in situ studies that cannot simulate the complex oral environment [162]. The results are not comparable with those found in clinical situations due to the difference in the erosive challenge, the small number of subjects and the short follow-up period.

## 4. Conclusions

Dental erosion is a common oral health problem that requires early intervention. Topical anti-erosive agents are commonly used for the nonrestorative management of dental erosion. These agents can be categorised as topical fluorides, calcium phosphate-based agents, organic compounds and other anti-erosive agents. Most topical anti-erosive agents are used to prevent dental erosion or control dental erosion in the early stages. Future research should be conducted to validate the clinical effectiveness of the anti-erosive agents and to develop topical agents with enhanced anti-erosive effects.

## Figures and Tables

**Table 1:** Summary of commercially available anti-erosive agents in the literature.

Agents	Concentration of Active Ingredients	Delivery System	Example of Product Names and Companies
NaF	1100 ppm F of NaF	Toothpaste	Colgate^®^ Total^®^, Colgate-Palmolive [33,43] Crest^®^ Cavity Protection, The Procter & Gamble [44]
	1450 ppm F of NaF	Toothpaste	Sensodyne^TM^ ProNamel^®^, GSK [18,19,21] Crest^®^ Decay Protection, The Procter & Gamble [41] Colgate^®^ Total^®^, Colgate-Palmolive [46]
	5000 ppm F of NaF	Toothpaste	Prevident 5000^®^, Colgate–Palmolive [20]
AmF + SnCl_2_	800 ppm Sn SnCl_2_ + 500 ppmF of NaF+ AmF	Mouthrinse	Elmex^®^ Erosion Protection dental rinse, GABA Schweiz, Colgate-Palmolive [12,50]
AmF + SnF_2_	250 ppm F of AmF + SnF_2_	Mouthrinse	Meridol^®^, Colgate-Palmolive [34]
	1450 ppmF of AmF + SnF_2_	Toothpaste	Meridol^®^, GABA Benelux [35]
SnF_2_	0.4–0.454% SnF_2_, 1100–1450 ppm F of SnF_2_	Toothpaste	Crest^®^ Pro HealthTM Advanced Gum Protection, The Procter & Gamble [33,42,43] Solidox, Lilleborg [51]
	1450 ppm F of NaF + SnF_2_	Toothpaste	Oral-B^®^ Pro-Expert Sensitive; The Procter & Gamble [21,47]
CPP-ACP	10% CPP-ACP	Gum	Trident Total^®^/Recaldent^®^, Trident [74]
	10% CPP-ACP	Cream	GC Tooth Mousse, GC Company [73,75,81] MI Paste, GC company [80]
	10% CPP-ACP with 900 ppm F of NaF	Cream	GC Tooth Mousse Plus, GC Company [81] MI Paste Plus, GC company [75,80]
	2% CPP-ACP + 22,600 ppm F of NaF	Varnish	MI varnish, GC Company [13]
CSSP	CSSP with 1450 ppm F of SMFP	Toothpaste	Regenerate^TM^ Enamel Science, Unilever Oral Care [83,86]
β-TCP	5% TCP + 22,600 ppm F of NaF	Varnish	Clinpro white varnish, 3M ESPE [13]
n-HAP	2.25–20% n-HAP	Toothpaste	Biorepair Sensitive Repair, Coswell [95] PrevDent, PrevDent International [90]
	1% n-HAP + 1450 ppm F of NaF	Toothpaste	ApaCare, Cumdente [90]
Arginine	1.5% Arginine + 1450 ppm F of SMFP	Toothpaste	∙ Colgate^®^ Maximum Cavity Protection, Colgate-Palmolive [115,117]
	8% Arginine + 1450 ppm F of SMFP	Toothpaste	Colgate Sensitive Pro-Relief^®^, Colgate-Palmolive [114,118]
Chitosan	0.5% Chitosan + 1400 ppm F AmF/NaF + 3500 ppm Sn SnCl_2_	Toothpaste	Elmex^®^ Erosionschutz and Elmex^®^ Erosion Protection, GABA Schweiz, Colgate-Palmolive [47]
CHX	0.2% Chlorhexidine	Mouthrinse	Periogard, Colgate-Palmolive [159] Klorhex, Drogsan Pharmaceutical [161]

NaF, sodium fluoride; SnF_2_, stannous fluoride; AmF, amine fluoride; SnCl_2_, stannous chloride; CPP-ACP, casein phosphopeptide amorphous calcium phosphate; CPP-ACFP, casein phosphopeptide amorphous calcium fluoride phosphate; CSSP, calcium silicate and sodium phosphate; β-TCP, β-tricalcium phosphate; F, fluoride; SMFP, sodium monofluorophosphate; ppm, parts per million; n-HAP, nano hydroxyapatite; CHX, chlorhexidine.

**Table 2:** Summary of the properties of topical anti-erosive agents in the literature.

	Form a Protective Layer	Modify Hydroxy-Apatite	Modify Salivary Pellicle	Neutralise Acid	Supply Mineral Ions	Inhibit MMPs
**Fluoride agents**						
NaF	√	√	√	×	×	√
AmF	√	√	?	×	×	?
SnF_2_	√	√	√	×	×	√
TiF_4_	√	√	?	×	×	?
SDF	√	√	?	√	×	√
**Calcium phosphate-based agents**						
CPP-ACP	√	×	√	×	√	×
CSSP	√	×	?	×	√	×
β-TCP	√	×	?	×	√	×
n-HAP	√	×	?	×	√	×
TMP/SHP	√	√	√*	×	√	×
LPP	√	×	√*	×	√	×
Pyrophosphate	√	√	?	×	√	×
Calcium lactate	√	×	?	×	√	×
**Organic compounds**						
PVM/MA	√	×	?	×	×	×
Carbopol	√	×	√*	×	×	×
PGA	√	×	√*	×	×	×
Arginine	√	×	?	×	×	×
Aspartame	?	×	?	×	×	×
CaneCPI-5	√	×	√	×	×	√
Casein	√	√	√	×	×	×
Chitosan	√	×	√	×	×	√
Palm oil	?	×	√	×	×	×
EGCG	√	×	√	×	×	√
Euclea natalensis	√	×	√	×	×	√
Proanthocyanidin	√	×	?	×	×	√
**Other agents**						
EBTiO_2_	√	×	?	×	√	×
Mg(OH)_2_	√	√	?	√	×	×
FeSO_4_	√	×	?	×	×	√
NaHCO_3_	?	×	?	√	×	×
CHX	√	×	?	?	×	√

√, positive impact; √*, negative impact; ×, no property; ?, unknown; NaF, sodium fluoride; SnF2, stannous fluoride; AmF, amine fluoride; TiF_4_, titanium tetrafluoride; SDF, silver diamine fluoride; CPP-ACP, casein phosphopeptide amorphous calcium phosphate; CPP-ACFP, casein phosphopeptide amorphous calcium fluoride phosphate; CSSP, calcium silicate and sodium phosphate; β-TCP, β-tricalcium phosphate; TMP, sodium trimetaphosphate; SHP, sodium hexametaphosphate; LPP, linear sodium polyphosphate; n-HAP, nano-hydroxyapatite; PVM/MA, polymethylvinylether-maleic anhydride; PGA, propylene glycol alginate; EGCG, epigallocatechin gallate; CaneCPI-5, sugarcane cystatin; EBTiO_2_, nano eggshell/titanium dioxide; Mg(OH)_2_, magnesium hydroxide; FeSO_4_, ferrous sulfate; NaHCO_3_, sodium bicarbonate; CHX, chlorhexidine.

## Data Availability

Not applicable.
